# Assessment of the T-SPOT.CMV interferon-γ release assay in renal transplant recipients: A single center cohort study

**DOI:** 10.1371/journal.pone.0193968

**Published:** 2018-03-20

**Authors:** Dimitrios Chanouzas, Alexander Small, Richard Borrows, Simon Ball

**Affiliations:** 1 Department of Nephrology, Queen Elizabeth Hospital Birmingham, Birmingham, United Kingdom; 2 Institute of Immunology and Immunotherapy, College of Medical and Dental Sciences, University of Birmingham, Birmingham, United Kingdom; 3 Institute of Translational Medicine, Birmingham, United Kingdom; University of Toledo, UNITED STATES

## Abstract

**Background:**

The measurement of CMV specific cellular immunity in organ transplant recipients could contribute additional acuity to serology based, CMV infection risk stratification, facilitating optimisation of immunosuppression and anti-viral prophylaxis.

**Methods:**

A pilot study of renal transplant recipient (RTR’s) responses in the T-SPOT.CMV ELISPOT based assay. 108 RTR’s were recruited 3 months post-transplantation, immediately prior to the cessation of stratified anti-viral prophylaxis, used in recipients from seropositive donors. RTR’s were monitored for CMV viremia and disease. Cellular responses to peptides derived from CMV IE1 and pp65 were measured, using the T-SPOT.CMV assay.

**Results:**

At recruitment, no CMV specific cellular immunity was detected by T-SPOT.CMV in CMV seronegative recipients (IE1 ≤ 1spot / 2.5x10^5^ PBMC’s; pp65 ≤ 3 spots / 2.5x10^5^ PBMC’s). At recruitment, CMV sero-positive recipients who made a robust response to both IE1 (>25 spots / 2.5x10^5^ PBMC’s) and pp65 (>50 spots / 2.5x10^5^ PBMC’s), were less likely to develop high level viremia than those who responded to one or neither antigen (0/28 vs 5/25; p<0.02).

**Conclusions:**

In CMV seronegative RTR’s, CMV specific cellular immunity measured by T-SPOT.CMV was not detected prior to cessation of anti-viral prophylaxis. This differs from recent reports of CMV specific cellular immunity in a proportion of CMV seronegative RTR’s, associated with protection from CMV infection. In seropositive RTR’s, a dual response to IE1 and pp65 at recruitment, was associated with protection from subsequent viremia. This suggests that assessing the diversity of response to CMV antigens, may enhance risk stratification in this group.

## Introduction

CMV disease is a major complication of organ transplantation, associated with increased morbidity, mortality and allograft loss [1]. It is possible to stratify the risk of post-transplant CMV disease using evidence of specific immunity in the recipient and latent infection in the donor. In practice, these are both inferred from CMV specific humoral immunity, the donor and recipient being referred to as seronegative (D- and R-) or seropositive (D+ and R+). The risk of CMV disease is highest in D+R- recipients, and this group has therefore been the focus of antiviral prophylaxis, in which there is clear evidence of benefit [2]. Although antiviral prophylaxis successfully suppresses early CMV disease, some recipients develop late disease after cessation of prophylaxis; at a time when follow-up is less frequent and presentation may therefore be delayed [1]. Evaluation of CMV disease risk beyond that achieved using donor and recipient CMV serostatus could further improve stratification of antiviral prophylaxis and immunosuppressive therapy. Since cellular immunity plays a pivotal role in controlling CMV infection, it is hypothesized that its quantification could enhance current risk stratification algorithms. For example, there is evidence that measures of serological and cellular immunity are discordant in some individuals. This provides a possible explanation for relative disease resistance or susceptibility in R- and R+ individuals respectively [3, 4].

The aim of this study was to pilot use of a new enzyme-linked immunospot (ELISPOT) based assay (T- SPOT^®^.CMV assay; Oxford Immunotec Ltd, Oxfordshire, UK) in RTR’s recruited 3 months post-transplantation, immediately prior to discontinuation of anti-viral prophylaxis, which was used in serologically defined high risk sub-groups. This assay measures the peripheral blood frequency of mononuclear cells producing γ-interferon to peptides derived from the complete sequence of two CMV antigens (immediate early 1 (IE-1) and phosphoprotein 65 (pp65)), with the potential to stimulate both class 1 and class 2 restricted responses. We evaluated samples for CMV specific cellular responses to determine whether detection of different CMV specific cellular responses predicts risk of infection or disease in RTR’s.

## Methods

The study was approved by the North-West (Lancaster) committee of the National Research Ethics Service, UK. Participants were recruited following written informed consent. None of the transplant donors were from a vulnerable population and all donors or next of kin provided written informed consent that was freely given. 115 adult recipients of solitary renal transplants were screened from day 75 post renal transplantation and 108 patients recruited for follow-up in a pilot, prospective, observational study of responses to CMV antigens in the T-SPOT.CMV test. All recipients received center standard immunosuppression which consisted of basiliximab induction followed by tacrolimus (target trough level 5–8 ng/ml, measured by liquid chromatography-tandem mass spectrometry), mycophenolate mofetil (2 g daily initially), and prednisolone (20 mg daily, reducing to 5 mg maintenance by 3 months after transplantation). CMV prophylaxis with 100 days of valganciclovir was used in D+R- and the D+R+ groups.

Baseline information was collected on the pre-transplant CMV serostatus of recipients and their donors, donor and recipient age and sex, cause of renal failure, HLA mismatch, source of transplant (living related, living unrelated, deceased donor following brain death, deceased donor following cardiac death). Postoperative events of delayed graft function (requirement for dialysis during the first postoperative week), biopsy-proven acute rejection and utilization of antiviral prophylaxis were collected over the first post-transplant year. Patients were excluded from recruitment, on the basis of anemia (Hemoglobin < 80g/L), if they had CMV disease since transplantation or if they intended to change long-term follow-up to another center (which was also the case for the 7 patients not included following screening due to a subsequent change of intention). No patients screened had a history of CMV disease by this timepoint.

Testing for CMV viremia was based on clinical indication with no real-time prospective assessment of CMV viremia performed. CMV disease was diagnosed according to international guidelines, based on one or more of the following in association with the finding of CMV viremia: fever, new-onset severe malaise, leukopenia, thrombocytopenia, hepatitis (alanine aminotransferase or aspartate aminotransferase levels greater than twice the upper limit of normal) and tissue-invasive disease proven by histology [1].

### Study assay

A T-SPOT.CMV test was undertaken on 5 occasions: 3, 4, 5, 6 and 12 months post-transplantation, initially prior to cessation of anti-CMV prophylaxis if that had been used. The CMV antigen specific response was reported as the frequency of spots per well containing 2.5 x 10^5^ PBMC’s. T-SPOT.CMV test samples were shipped on the same day as collection to Oxford Diagnostic Laboratories (Abingdon, UK), where the assay was performed within 32 hours of blood draw as per the validated test protocol. Results were excluded from analyses if the nil control was > 10 or if the PHA positive control failed to stimulate a response > 10 spots per well. Laboratory personnel were blinded to the patients’ clinical information. Clinical personnel were blinded to the T-SPOT.CMV result. A spot count reported as > 10 well is defined as positive by the manufacturer.

### Other assays

A quantitative nucleic acid test (QNAT) was undertaken at 3, 4, 5, 6, 9 and 12 months post-transplantation (Abbott RealTime CMV amplification kit and m2000 system (Ref 5N2390)). The lower limit of detection was 20 copies/mL and the lower limit of quantification was 200 copies/mL of plasma.

CMV serostatus was determined 12 months post-transplantation in patients previously determined to be seronegative prior to transplantation (Roche Elecsys CMV IgG assay (Ref 04784596) on a Roche modular E170). All other routine clinical assays were undertaken in the Department of Pathology at Queen Elizabeth Hospital Birmingham. The results of all assays were reported to the research team after study completion. High level viremia was defined as >5000 copies/ml [5].

### Statistical analysis

SPSS Statistics Version 21 (IBM, Armonk, USA) and Prism Versions 5 and 6 (GraphPad, La Jolla, USA) were used. Data were assessed for normality using the D’Agostino & Pearson omnibus normality test. Where assumptions of normality were not valid, data were analysed nonparametrically and median with interquartile range reported. Correlations were assessed with Pearson’s correlation, unpaired data with student’s t test (with Welch’s correction for unequal variances if appropriate) or the Mann Whitney U test depending on normality assumptions and paired data with paired t test or Wilcoxon matched-pairs signed rank test. Comparisons between categorical variables were assessed with Fisher’s exact test. All statistical analyses were two-tailed.

## Results

### Clinical outcomes and detection of viremia

The demographic and clinical variables of patients recruited to the study are summarized in Table 1. Clinically indicated CMV QNAT was available to clinicians as part of standard care, whilst on per protocol samples, CMV QNAT was undertaken at the end of the study.

**Table 1:** Demographic and clinical variables.

**Recipient age / yrs (median (IQR)**	49 (37–61)
**Male recipients, *n* (%)**	64 (59)
**Recipient ethnicity, *n***	
White	75
Indo-Asian	23
African-Caribbean	7
Other	3
**Cause of renal failure, *n* (%)**	
Glomerular	22 (20)
Hereditary/cystic	25 (23)
Diabetes	7 (6)
Vascular	24 (22)
Interstitial	7 (6)
Other	23 (21)
Donor age (yr)	48.9
**Transplant source, *n* (%)**	
Deceased donor	
*DBD*	49 (45.4)
*DCD*	8 (7.4)
Live donor	51 (47.2)
**Donor-recipient HLA mismatch**	
HLA-A	1.1
HLA-B	1.0
HLA-DR	0.7
**Donor-recipient CMV serostatus**	
D—R—	30
D—R+	25
D+R+	32
D+R—	21
**Day recruited post-transplantation (days) (interquartile range)**	84 (79–91)
**Immunosuppression on recruitment**	
(Basiliximab induction)	(108)
Tacrolimus	106
MMF	98
Corticosteroid	108
**Biopsy proven acute rejection**	9
**Patient survival at 9 months post-recruitment (1 year post transplantation), n (%)**	108 (100)
eGFR (ml/min) (SD) 12 months post transplantation in surviving transplants	52 (30)
Graft survival at 9 months post recruitment (1 year post-transplant) n (%)	107 (99)
CMV disease at 12 months (5 D+R-, 1 D+R+, 1 D-R+) n	7
CMV QNAT > 5000 copies /mL at any time-point, n	12

Amongst 30 D-R- recipients, no cases of CMV disease or viremia were detected.

Amongst 25 D-R+ recipients, 1 patient developed CMV colitis. On per-protocol samples, 2 others were found to have high level viremia (>5000 copies/ml) on month 5 or 6 samples, without CMV disease. 10 others had low level viremia detected on at least one occasion (maximum 742 copies/ml).

Amongst the 32 D+R+ recipients, 1 patient developed CMV colitis. On per-protocol samples, 2 others were found to have high level viremia on the month 5 or 6 samples, without CMV disease. 12 others had low level viremia detected on at least one occasion (maximum 940 copies/ml).

Amongst 21 D+R- recipients, 3 were diagnosed with CMV colitis and 2 CMV syndrome between month 5 and 6 post-transplantation. On per-protocol samples, one other recipient was found to have high level viremia (61,865 copies/ml) at month 6. 3 others had low level viremia detected on at least one occasion.

In the D-R+, D+R+, D+R- population, there were therefore 37/78 patients who were found to be viremic on at least one occasion during 9 months of follow-up, despite the fact that QNAT testing occurred no more often than monthly.

There were three patients who developed significant complications associated with other herpes viruses: one patient varicella zoster, one patient varicella retinitis and one patient post-transplant lymphoproliferative disorder.

### T-SPOT.CMV test at recruitment

At recruitment (a median of 84 days post-transplantation), only one negative control contained more than 1 spot (D-R+ recipient that failed negative control). The median positive control response to PHA was 253 (IQR 105–348) spots per well (2.5 x 10^5^ PBMC’s). The PHA positive control was failed by one D+R+ and one D-R+ recipient. One recruitment sample was unavailable for a D+R+ recipient.

**Fig 1:**
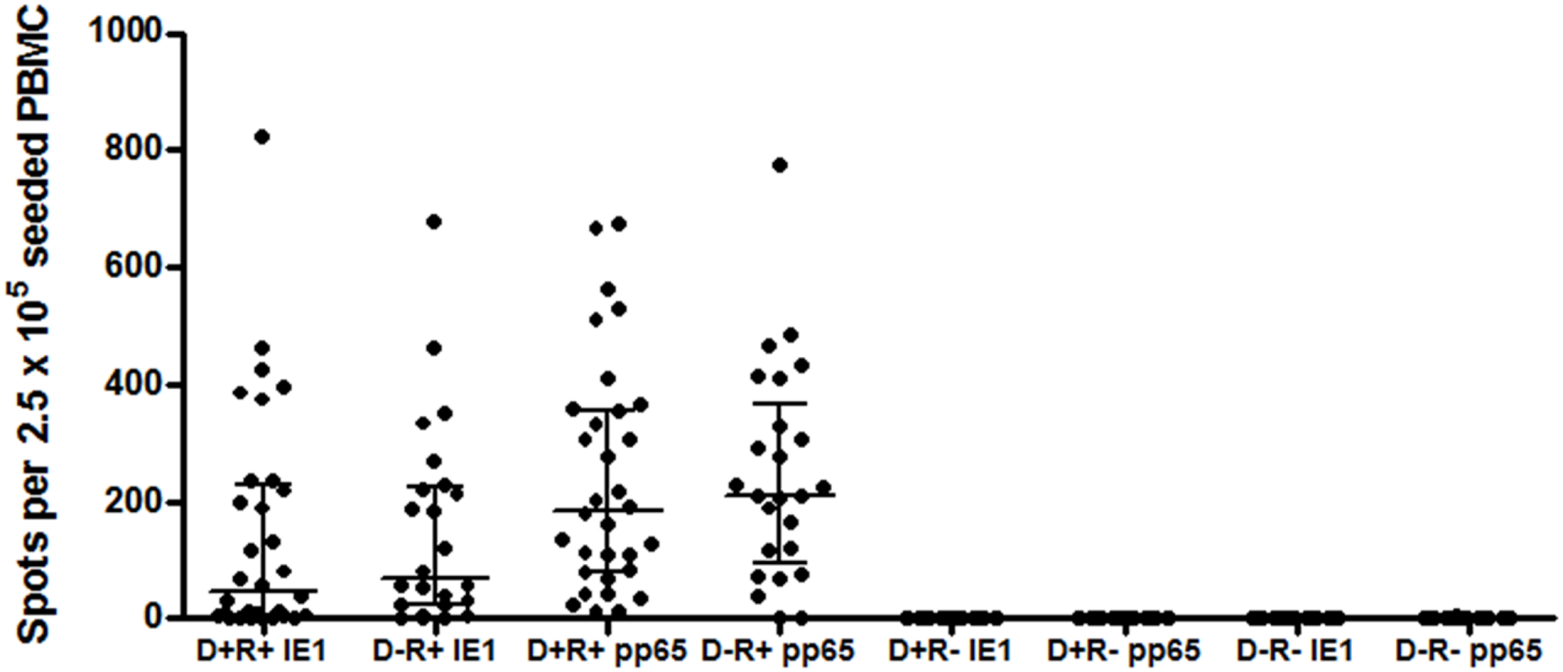
T-SPOT.CMV responses in renal transplant recipients at recruitment. T-SPOT.CMV test undertaken in renal transplant recipients at the time of study recruitment (3 months post-transplantation) prior to cessation of anti-CMV prophylaxis. The IE1 and pp65 specific response, reported as the frequency of spots per well containing 2.5 x 10^5^ PBMC’s, are shown. In D+R+ and in D-R+ RTR’s the frequency of response to pp65 derived peptides was significantly higher compared to IE1 derived peptides (p<0.05). There was no significant difference in the frequency of response to pp65 and IE1 between D+ and D- individuals (p = 0.43 for pp65 and p = 0.59 for IE1).

The responses to IE1 and pp65 in the T-SPOT.CMV assay at recruitment, stratified by donor and recipient CMV serostatus, are shown in Fig 1. At recruitment, the T-SPOT.CMV assay was negative in all R- RTR’s (IE ≤ 1spot / well (2.5 x 10^5^ PBMC’s); pp65 well ≤ 1spot / well (2.5 x 10^5^ PBMC’s).

**Fig 2:**
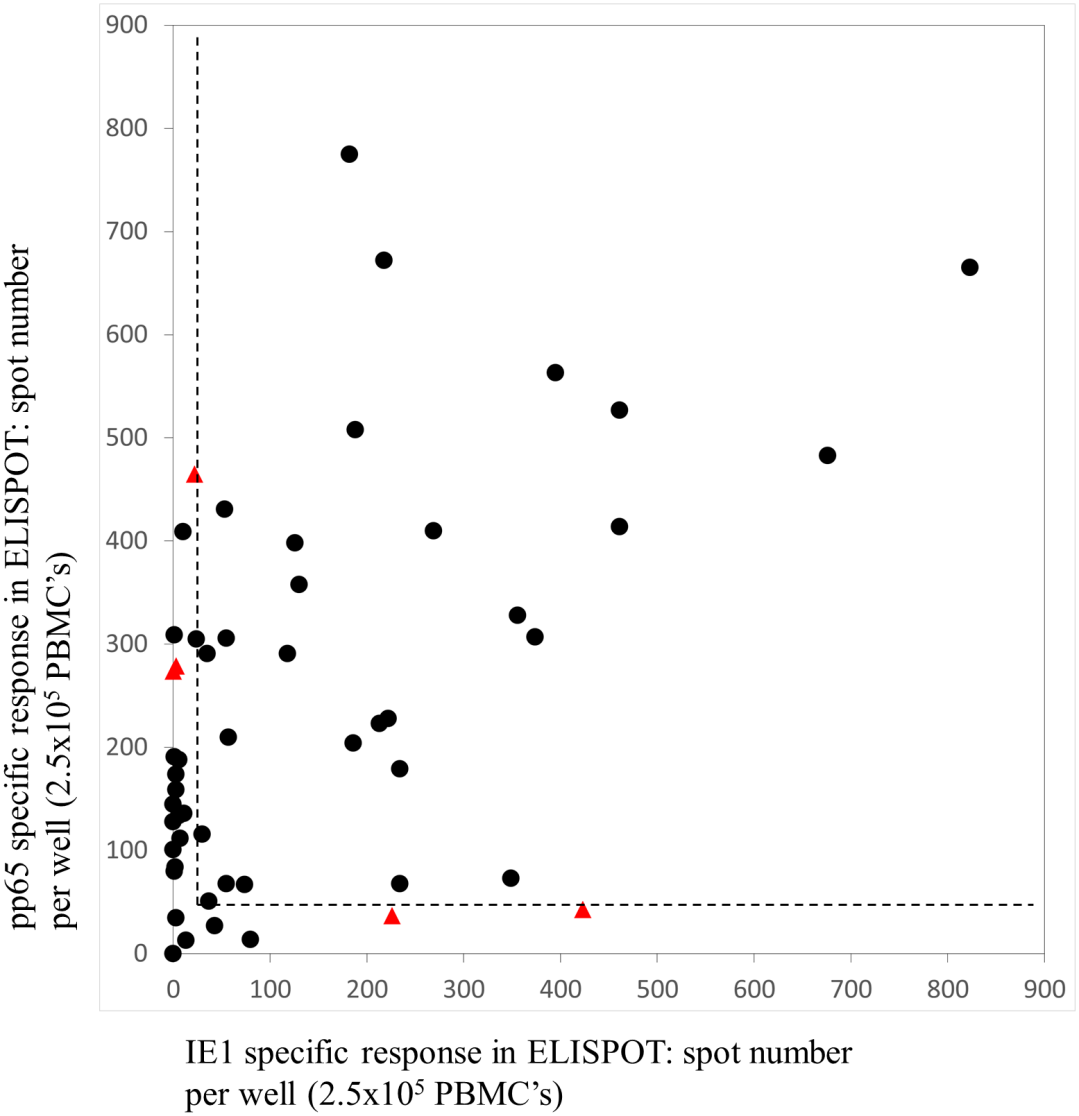
T-SPOT.CMV responses to pp65 and IE1 in R+ renal transplant recipients at recruitment. T-SPOT.CMV test undertaken 3 months post-transplantation in R+ renal transplant recipients. The pp65 and IE1 specific response, reported as the frequency of spots per well containing 2.5 x 10^5^ PBMC’s, are shown on the ordinate and abscissa respectively. Responses to pp65 and IE1 were significantly correlated (R^2^ = 0.24, p<0.001). The 28 recipients who made robust cellular responses to both pp65 and IE1 (defined as > 50 spots for pp65 and > 25 spots for IE1; limits displayed as dotted line) were less likely to subsequently develop high level CMV viremia (>5000 copies/ml, with or without evidence of CMV disease) compared to all others (0/28 vs 5/25; p<0.02; Fisher’s exact). RTR’s subsequently developing high level CMV viremia are identified by a triangle and all others by a circle.

In the 53 R+ RTR’s that passed control, the frequency of response to IE1 and pp65 were widely distributed (IE1 median = 56, IQR 6–256 spots/well; pp65 median = 204, IQR 80–356 spots/well). There was no difference between those who received a kidney from D+ or D- donors (p>0.4). In the T-SPOT.CMV assay at recruitment, the frequency of response to both IE1 (abscissa) and pp65 (ordinate) in R+ RTR’s shown in Fig 2, correlate significantly (p<10^−3^) but with a low coefficient of determination (R^2^ = 0.24).

In haematopoietic stem cell transplantation (HSCT) Nesher and colleagues recently proposed thresholds for recipients’ T- SPOT.CMV responses of 50 spots per well for IE1 and 100 spots per well for pp65 [6]. This was based upon protection from CMV infection in the absence of graft vs. host disease. Applying these thresholds, 21/53 R+ RTR responded to both antigens, a lower proportion response rate than defined in HSCT recipients by Nesher and colleagues. We chose a lower threshold of 25 spots per well (2.5 x 10^5^ PBMC’s) for IE1 and 50 spots per well (2.5 x 10^5^ PBMC’s) for pp65, to dichotomize our population, which increased the number defined as responding to both antigens to 28/53. Using these thresholds (shown by dotted lines in Fig 2), CMV disease or viremia defined by a QNAT >5000 copies/ml, was less frequent in R+ RTR’s in whom a response to both IE1 and pp65 antigens was detected at recruitment, compared to those without a response to both IE1 and pp65 (0/28 vs 5/25; p<0.02; Fisher’s exact test; cases of high level viremia shown as a triangle and all others by a circle in Fig 2). This finding was not changed by reducing the QNAT threshold for viremia to as low as 1000 copies/ml [5, 7].

### T-SPOT.CMV and CMV specific IgG in D+R- RTR’s up to 12 months post-transplantation

In the 9 D+R- RTR’s in whom viremia was detected during follow-up the T-SPOT.CMV assay was always negative before the detection of viremia (S1 Table). The T-SPOT.CMV assay became positive either at the time of detection of viremia or in the subsequent sample.

By 12 months post transplantation, of 5/21 D+R- recipients diagnosed with CMV disease, 3 made a robust response to both antigens as defined above (>25 spots per well for IE1 and >50 spots per well for pp65) and two did not (IE1 = 1 and 0 spot / well and pp65 = 9 and 47 spots/well). These two ‘poor responders’ had persistent low-level viremia at 12 months post-transplantation whereas the other 3 did not. Amongst the 4 D+R- RTR’s who were viremic without symptoms, 3 made a robust response to both antigens 12-month post-transplantation (>25 spots per well for IE1 and >50 spots per well for pp65) but 1 responded only to pp65 (IE1 = 2 spot / well and pp65 = 81 spots/well). No viremia was detected by 12 months in these 4 asymptomatic RTR’s.

Amongst the 9/21 D+R- RTR’s in whom CMV viremia was detected, all seroconverted when retested 12 months post-transplantation (data unavailable in one). Of the remaining 12 patients in whom no CMV was detected by QNAT, two seroconverted by 12-months. These two had no T-SPOT.CMV response detected on any sample. There was no T-SPOT.CMV response, viremia or humoral response detected in the other 10 patients on any sample.

### T-SPOT.CMV and CMV specific IgG at 12 months in D-R- transplant recipients

Amongst 30 D-R- recipients there was no evidence of CMV infection, T-Spot.CMV response or sero-conversion by 12 months post-transplantation.

## Discussion

This study pilots the T-SPOT.CMV interferon-γ release assay (IGRA) in renal transplantation, as a potential adjunct to serologically based CMV disease risk stratification. Precise attribution of risk could contribute to targeted anti-viral and immunosuppressive strategies to improve outcomes.

In our study, CMV seronegative RTR’s recruited prior to cessation of antiviral prophylaxis, did not respond to IE1 or pp65 derived peptides in the T-SPOT.CMV IGRA, and could not therefore contribute to the evaluation of risk in this group. This differs from reports in which evidence of CMV specific cellular immunity is found in a proportion of seronegative individuals. These reports include that by Lucia and colleagues using IE1 and pp65 derived peptides in a γ-interferon ELISPOT [3], by Manuel and colleagues using IE1 and pp65 derived peptides in cell culture supernatant interferon-γ assay (Quantiferon-CMV^®^) [4] and by Banas and colleagues using a γ-interferon ELISPOT to whole protein (T-Track-CMV^®^) [8]. In contrast, Abate and colleagues have reported that cellular immune responses to pp65 were concordant with CMV serostatus in renal transplant recipients and in healthy women [9, 10].

As in our study, Lucia and colleagues measured γ-interferon ELISPOT responses to a set of overlapping 15mer peptides derived from full length pp65 and IE1. Subjects were tested prior to transplantation. They found approximately 30% of R- recipients responded to these antigens. The distribution of responses of R+ recipients in their assay (IE1 45±95, pp65 120 ± 181 (mean ± SD) spots per 3x10^5^ PBMC’s) are not obviously different from those observed with the T-SPOT.CMV IGRA, suggesting that these contradictory findings do not reflect simple differences in sensitivity of the respective assays. There are differences in methodology such as their use of frozen and thawed PBMC’s and in the assay of CMV serostatus, although these seem unlikely to account for the observed differences. Specifically, inter-rater agreement across modern commercial assays of CMV serology are high, albeit that their performance may require re-evaluation, in light of Lucia’s finding CMV specific B lymphocytes in R- recipients.

Perhaps the most significant difference is that the IGRA was performed post-transplantation in our study, when recipients were immunosuppressed. It is therefore possible that CMV specific T cell reactivity in R- recipients, was reduced below the level of detection. Since in Lucia’s study [3], R- IGRA responders did so with significantly lower frequency than R+ IGRA responders, the R- response may be more sensitive to the effects of immunosuppression. Future studies might therefore define conversion and reversion rates in patients assessed pre- and post-transplantation.

The study by Manuel and colleagues [4] recruited patients at a similar time-point to ours. If later time-points were also included, 25% of R- recipients were found to respond in the Quantiferon-CMV^®^ IGRA and they were observed to have a lower incidence of CMV disease over the first post-transplant year. However, this included a proportion of recipients who developed an IGRA response following the cessation of prophylaxis, which might then include patients who develop cellular immunity following transient asymptomatic viremia: a finding in 4/21 D+R- recipients in our cohort, after prophylaxis was stopped.

A recent study by Banas and colleagues [8] demonstrates significant variation in attribution of CMV specific cellular immunity according to methodological platform, above and beyond the question of the temporal relationship of testing to transplantation, immunosuppression and prophylaxis. Using 3 different assays defining CMV specific immunity in seronegative dialysis patients they identified cellular responses in 21/57, but these were discordant in all but one individual. The method and timing of assays determining CMV specific cellular immunity for clinical purposes therefore remains a matter for further assessment.

Our observation that two D+R- recipients seroconverted without evidence of a cellular response or viremia may relate to infrequent QNAT testing. They could have been transiently viremic without symptoms. On the other hand, in the 4 recipients in whom viremia was documented and who seroconverted, there was also a T-SPOT.CMV response.

In the R+ cohort the CMV disease event rate was low, possibly because prophylaxis was used in the D+R+ group, a strategy instituted at our centre following analysis of an earlier series [11]. In light of this low event rate, high-level CMV viremia was used as a surrogate outcome measure, which was subsequently related to the initial T-SPOT.CMV result. Any threshold for asymptomatic viremia is to an extent arbitrary; we based our definition of high-level viremia on the influential analysis by Humar and colleagues [5], although this does relate to an era before valganciclovir based CMV prophylaxis. Our data suggest that in R+ recipients, a response to both IE1 and pp65 at 3 months post-transplantation is associated with relative protection from subsequent high-level viremia. This observation is also consistent with finding that in D+R- recipients who developed CMV disease following the cessation of anti-viral prophylaxis, clearance of viremia occurred only in those who responded to both antigens by 12 months post-transplantation, although the numbers are too small to make any definitive conclusion. An important caveat is that this is a pilot study and any such conclusion requires validation using the same IGRA and definitions of response. This may be possible following completion of the PROTECT study (NCT02382211).

In CMV infected individuals, responses to IE-1 and pp65 dominate the CD8+ T cell response, although there is significant inter-individual variation. In R+ recipients, some reports suggest that the correlation between CMV disease and IGRA responses to pp65 and IE1, is insufficient provide any clinical utility [3, 9]. They did not however analyse the combined response to both pp65 and IE1. Interestingly, in studies of solid organ transplantation it is primarily the response to IE1 that has been linked to disease-protection [12, 13]. A potentially related finding in our study is that the regression line between pp65 and IE1 T-SPOT.CMV responses, has a highly positive intercept on the pp65 axis. That is, a high IE1 response approximates to a high response to both pp65 and IE1.

Although the literature in clinical transplantation is dominated by these high frequency CD8+ T cell responses to pp65 and IE1 [14], there is increasing evidence that responses to other CMV antigens [15–17], by different arms of the immune system may be relevant to the control of CMV infection and its pathogenesis [18–24]. Predicting disease protection from responses to single antigens may therefore have intrinsic limitations [17]. The T-SPOT.CMV IGRA uses these two antigens, represented by a full range of overlapping peptides, with potential to stimulate both class I and class II restricted T cell responses. It is therefore possible that our preliminary observation of an apparent protection from high level viremia, in R+ recipients who respond to both antigens, is the herald of a diverse CMV specific immune response.

In summary, in renal transplant recipients recruited 3 months post-transplantation, we found no evidence of CMV specific cellular immunity using the T-SPOT.CMV IGRA in those without humoral immunity in the Roche Elecsys CMV IgG assay. A dual response to both pp65 and IE1 may identify R+ renal transplant recipients relatively protected from CMV reactivation, a finding that warrants further investigation. Asymptomatic, transient, low level viremia in D+R- recipients was associated with the subsequent acquisition of a T-SPOT.CMV IGRA response and with seroconversion.

## Supporting information

S1 Table T-SPOT.CMV in D+R- renal transplant recipients with detectable viremia. (DOCX)

## Abbreviations

CMV: cytomegalovirus
D-: donor CMV seronegative
D+: donor CMV seropositive
ELISPOT: enzyme linked immunospot
IE-1: immediate early 1
IGRA: interferon-γ release assay
pp65: phosphoprotein 65
R-: recipient CMV seronegative
R+: recipient CMV seropositive
RTR: renal transplant recipients
