# Supplementary material for: Assessment of the T-SPOT.CMV interferon-γ release assay in renal transplant recipients: A single center cohort study
Source: PLoS One. 2018 Mar 20;13(3):e0193968. doi: 10.1371/journal.pone.0193968 (PMC5860728; doi:10.1371/journal.pone.0193968)
Supplement: S1 Table — (DOCX) [file pone.0193968.s001.docx]

**Supplementary Table 1**

| **Case** |  | **Month 3 (recruitment)** | **Month 4** | **Month 5** | **Month 6** | **Month 9** | **Month 12** |
| --- | --- | --- | --- | --- | --- | --- | --- |
| 1 | CMV QNAT | ND | ND | 189669 | 1400 | 34 | ND |
|  | T-SPOT.CMV pp65 | 0 | 0 | 33 | 165 |  | 212 |
|  | T-SPOT.CMV IE1 | 1 | 1 | 21 | 136 |  | 194 |
|  |  |  |  |  |  |  |  |
| 9 | CMV QNAT | ND | 222 | 5637 | 23 | ND | ND |
|  | T-SPOT.CMV pp65 | 1 | 18 | 13 | 160 |  | 363 |
|  | T-SPOT.CMV IE1 | 1 | 291 | 86 | 88 |  | 84 |
|  |  |  |  |  |  |  |  |
| 14 | CMV QNAT | ND | ND | 43614 | 78 | ND | ND |
|  | T-SPOT.CMV pp65 | 0 | 0 | 0 | 0 |  | 103 |
|  | T-SPOT.CMV IE1 | 0 | 0 | 0 | 6 |  | 114 |
|  |  |  |  |  |  |  |  |
| 16 | CMV QNAT | ND | ND | ND | 141016 | 1030 | 271 |
|  | T-SPOT.CMV pp65 | 0 | 0 | 0 | 0 |  | 0 |
|  | T-SPOT.CMV IE1 | 0 | 0 | 0 | 0 |  | 47 |
|  |  |  |  |  |  |  |  |
| 27 | CMV QNAT | ND | ND | 25877 | 25215 | 116 | 28 |
|  | T-SPOT.CMV pp65 | 0 | 0 | 0 | 0 |  | 1 |
|  | T-SPOT.CMV IE1 | 1 | 0 | 1 | 1 |  | 9 |
|  |  |  |  |  |  |  |  |
| 10 | CMV QNAT | ND | ND | ND | 2101 | ND | ND |
|  | T-SPOT.CMV pp65 | 0 | 0 | 0 | 0 |  | 177 |
|  | T-SPOT.CMV IE1 | 0 | 0 | 0 | 6 |  | 132 |
|  |  |  |  |  |  |  |  |
| 31 | CMV QNAT | ND | ND | ND | 901 | 45 | ND |
|  | T-SPOT.CMV pp65 | 1 | 1 | 1 | 11 |  | 242 |
|  | T-SPOT.CMV IE1 | 0 | 0 | 0 | 221 |  | 202 |
|  |  |  |  |  |  |  |  |
| 62 | CMV QNAT | ND | ND | ND | 61865 | 676 | ND |
|  | T-SPOT.CMV pp65 | 0 | 0 | 0 | -- |  | 73 |
|  | T-SPOT.CMV IE1 | 0 | 0 | 1 | -- |  | 102 |
|  |  |  |  |  |  |  |  |
| 63 | CMV QNAT | ND | ND | ND | ND | 570 | ND |
|  | T-SPOT.CMV pp65 | 0 | -- | 0 | 0 |  | 2 |
|  | T-SPOT.CMV IE1 | 1 | -- | 1 | 0 |  | 81 |

CMV QNAT expressed as viral copies / mL

T-SPOT.CMV expressed as number of spots / 2.5 x 10^5^ PBMC

ND = not detected

Cases 1, 9, 14, 16, 27 had CMV disease

Cases 10, 31, 62, 63 had asymptomatic viremia only

For cases 62 and 63 there was no available T-SPOT.CMV sample at month 6 and month 4 respectively
